# A New Strategy for Efficient Retrospective Data Analyses for Designer Benzodiazepines in Large LC-HRMS Datasets

**DOI:** 10.3389/fchem.2022.868532

**Published:** 2022-05-19

**Authors:** Meiru Pan, Brian Schou Rasmussen, Petur Weihe Dalsgaard, Christian Brinch Mollerup, Marie Katrine Klose Nielsen, Michael Nedahl, Kristian Linnet, Marie Mardal

**Affiliations:** ^1^ Department of Forensic Medicine, University of Copenhagen, Copenhagen, Denmark; ^2^ Department of Pharmacy, The Arctic University of Norway, Tromsø, Norway

**Keywords:** retrospective screening, HRMS, designer benzodiazepines, cheminformatics, new psychoactive substances, drug screening, LC-QTOF

## Abstract

The expanding and dynamic market of new psychoactive substances (NPSs) poses challenges for laboratories worldwide. The retrospective data analysis (RDA) of previously analyzed samples for new targets can be used to investigate analytes missed in the first data analysis. However, RDA has historically been unsuitable for routine evaluation because reprocessing and reevaluating large numbers of forensic samples are highly work- and time-consuming. In this project, we developed an efficient and scalable retrospective data analysis workflow that can easily be tailored and optimized for groups of NPSs. The objectives of the study were to establish a retrospective data analysis workflow for benzodiazepines in whole blood samples and apply it on previously analyzed driving-under-the-influence-of-drugs (DUID) cases. The RDA workflow was based on a training set of hits in ultrahigh-performance liquid chromatography–quadrupole time-of-flight–mass spectrometry (UHPLC-QTOF-MS) data files, corresponding to common benzodiazepines that also had been analyzed with a complementary UHPLC–tandem mass spectrometry (MS/MS) method. Quantitative results in the training set were used as the true condition to evaluate whether a hit in the UHPLC-QTOF-MS data file was true or false positive. The training set was used to evaluate and set filters. The RDA was used to extract information from 47 DBZDs in 13,514 UHPLC-QTOF-MS data files from DUID cases analyzed from 2014 to 2020, with filters on the retention time window, count level, and mass error. Sixteen designer and uncommon benzodiazepines (DBZDs) were detected, where 47 identifications had been confirmed by using complementary methods when the case was open (confirmed positive finding), and 43 targets were not reported when the case was open (tentative positive finding). The most common tentative and confirmed findings were etizolam (*n* = 26), phenazepam (*n* = 13), lorazepam (*n* = 9), and flualprazolam (*n* = 8). This method efficiently found DBZDs in previously acquired UHPLC-QTOF-MS data files, with only nine false-positive hits. When the standard of an emerging DBZD becomes available, all previously acquired DUID data files can be screened in less than 1 min. Being able to perform a fast and accurate retrospective data analysis across previously acquired data files is a major technological advancement in monitoring NPS abuse.

## 1 Introduction

Benzodiazepines are a group of substances widely prescribed for the treatment of anxiety, insomnia, muscle spasms, alcohol withdrawal, and epilepsy. Benzodiazepines were the group of medicinal drugs most frequently detected above the legal threshold in driving-under-the-influence-of-drugs (DUID) cases in Denmark from 2015 to 2019 (Simonsen et al., 2022). In recent years, there has been an increase in designer and uncommon benzodiazepines (DBZDs) on the illicit drug market (Orsolini et al., 2020). In 2019, 1,240 seizures of DBZDs were reported to the European Union Early Warning System (European Monitoring Centre for Drugs and Drug Addiction (EMCDDA), 2021a). In February 2021, 30 new benzodiazepines were monitored by the European Monitoring Centre for Drugs and Drug Addiction (EMCDDA) (European Monitoring Centre for Drugs and Drug Addiction (EMCDDA), 2021b). The group of DBZDs includes former drug lead candidates, drugs chemically modified to circumvent drug legislation, and drugs not covered by legislation in other countries. The co-consumption of DBZDs with other psychoactive substances like alcohol and opioids is common, which amplifies the risks of serious adverse events (European Monitoring Centre for Drugs and Drug Addiction (EMCDDA), 2018). DBZDs are commonly used as recreational drugs and reported in drug-facilitated crime and DUID cases (Valen et al., 2017; Bertol et al., 2018; Pan et al., 2019; Zawilska and Wojcieszak, 2019).

The emergence of new psychoactive substances (NPSs) does not only pose a considerable health concern but also pose a challenge to forensic toxicology laboratories. The emerging substances are often not included in the toxicology screening method, and the reference standards might not be commercially available. Sharing analytical data between laboratories can reduce the time elapse from the initial detection of an NPS until laboratories start screening for the compounds (Mardal et al., 2019). However, some lag time is inevitable. Various methods have been published to detect DBZDs in biological specimens: immunological techniques (Pettersson et al., 2016), gas chromatography–mass spectrometry (GC-MS) (Inoue et al., 2000), and liquid chromatography–mass spectrometry (LC-MS) (Banaszkiewicz et al., 2020). The application of high-resolution mass spectrometry (HRMS) has been widely used as a screening method, offering high sensitivity and selectivity, and the flexible addition of new compounds to the screening libraries.

Retrospective data analysis (RDA) studies for NPSs in forensic cases have reported or looked for new findings that were not detected during the initial analyses (Noble et al., 2018; Partridge et al., 2018; Fels et al., 2020; Gundersen et al., 2020). When ultrahigh-performance liquid chromatography coupled with quadrupole-time-of-flight-mass-spectrometry (UHPLC-QTOF-MS) is performed with data-independent acquisition (DIA), the full-spectrum data of both precursor ions and fragment ions are generated in a single run together with chromatographic retention data. In this way, a retrospective analysis of recorded data is possible to look for new targets without reanalyzing the samples. RDA in historic QTOF-MS data is a way to monitor to what extent this group of compounds was not detected when the sample was originally analyzed. However, RDA has been unsuitable for routine evaluation because reprocessing and reevaluating large numbers of forensic samples is highly work- and time-intensive.

**FIGURE 1:**
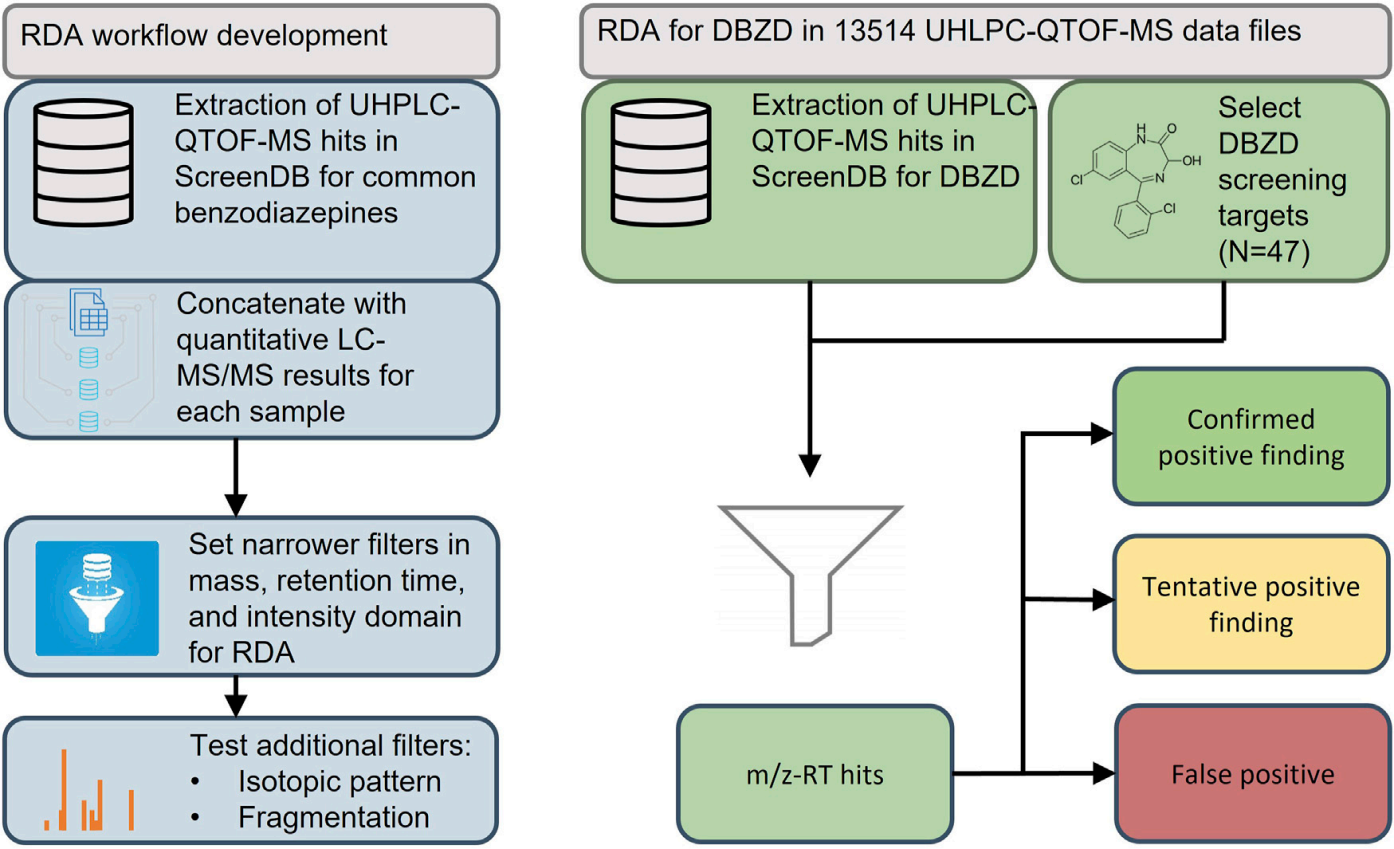
Workflow for the development and application of a retrospective data analysis for designer benzodiazepines (DBZDs) in whole blood. QTOF-MS, quadrupole time of flight–mass spectrometry; LC-MS/MS, liquid chromatography–tandem mass spectrometry; RDA, retrospective data analysis; RT, retention time.

The aim of this study was to use a new strategy to develop fast and efficient RDA workflows tailored for groups of NPSs based on historic data from standard forensic cases. The objective was to develop an RDA workflow tailored to benzodiazepines and use this to identify DBZDs in blood samples from DUID cases analyzed from 2014 to 2020. The study focused on analytes that had been analyzed using the UHPLC-QTOF-MS screening instrument as standards or drug seizures and covered DBZDs and less frequently prescribed benzodiazepines. An outline of the study is presented in Figure 1.

## 2 Materials and Methods

### 2.1 Chemicals and Reagents

The reference standards were obtained from BD Biosciences (Franklin Lakes, NJ, United States), Cayman Chemicals (Ann Arbor, MI, United States), Cerilliant (Round Rock, TX, United States), Chiron AS (Trondheim, Norway), LGC Standards (Wesel, Germany), Lipomed AG (Arlesheim, Switzerland), Sigma-Aldrich (Schnelldorf, Germany), Toronto Research Chemicals (North York, ON, Canada), USP (Rockville, MD, United States), Wyeth Pharmaceuticals LLC (Collegeville, PA, United States), and standards donated by the Slovenian National Forensic Laboratory. The internal standards (ISs) amphetamine-d5, diazepam-d5, methadone-d3, mianserin-d3, and morphine-d6 were purchased from Cerilliant (Round Rock, TX, United States). Acetonitrile, ammonium acetate, ammonium formate, formic acid, and methanol were of LC-MS grade and obtained from Fisher Scientific (Loughborough, United Kingdom).

### 2.2 Samples

In this retrospective study, authentic whole blood samples from 13,514 UHPLC-QTOF-MS data files from DUID cases analyzed from October 2014 to October 2020 were evaluated for DBZDs. All biological samples were preserved with 1% sodium fluoride. As part of the standard forensic analyses, the samples were subjected to protein precipitation and subsequently analyzed by UHPLC-QTOF-MS (general screening) supplemented with various LC-MS/MS approaches as previously described (Mollerup et al., 2017; Hansen et al., 2021). The details for the UHPLC-QTOF-MS and LC-MS/MS methods for quantitation of frequently detected benzodiazepines are described in Supplementary Information S1 (SI1). In brief, the UHPLC-QTOF-MS general screening of DUID cases relies primarily on targeted screening. Target identifications were performed by matching candidate components with our in-house library, which contains molecular formula and structure and expected RT, and most of them have exact masses of product ions. The in-house library is continuously updated when we find new substances from drug seizures, and currently, it contains 5,409 compounds. If a DBZD was identified as a part of the DUID case evaluation, the finding would be confirmed and quantified by using a complementary UHPLC-MS/MS analytical method, based on the requirement of the case.

### 2.3 Instrument Conditions

#### 2.3.1 UHPLC-QTOF-MS Conditions

LC was performed using an ACQUITY UPLC system from Waters Corporation (Milford, MA, United States) with an ACQUITY UPLC HSS C_18_ column (150 mm × 2.1 mm, 1.8 μm). HRMS was performed using Xevo G2-S QTOF (Waters MS Technologies, Manchester, United Kingdom). The system operation and data analysis were performed with UNIFI (Waters Corporation, Milford, MA, United States). Data were acquired in the positive ionization mode with DIA and elevated collision energy (MS^E^) with a resolution of 32,500 FWHM. Leucine enkephalin (m/z 556.2766) was used as the lock mass. Mass calibration was performed weekly with sodium formate solution in propanol:water (90:10, v/v).

#### 2.3.2 UPLC-MS/MS Conditions

Quantitative analysis for DBZDs and BZD was performed with the same system as described in a former study (Hansen et al., 2021) using an ACQUITY UPLC coupled to an ACQUITY TQ-S or TQD triple quadrupole mass spectrometers (Waters, Milford, MA, United States). Chromatographic separation was achieved using an Acquity UPLC BEH C_18_ (1.7 µm 2.1 × 50 mm) for BZD and (1.7 µm 2.1 × 100 mm) for DZBD, respectively. The MS system was in the positive electrospray ionization mode (ESI^+^) with multiple reaction monitoring transitions. For transition information and other operation conditions, see Supplementary Information S2. The method for BZD was fully validated (Hansen et al., 2021), and the validation parameters for the analytical runs with DZBZ cases are presented in Supplementary Information S3.

### 2.4 Data Processing and Analysis

#### 2.4.1 UPLC-QTOF-MS Screening of Driving-Under-the-Influence-of-Drugs Samples

Previously componentized data from the general screening were used for the RDA. UNIFI software performed componentization with 3D peak detection, high-collision energy channel–low-collision energy channel association (Mollerup et al., 2017). After the evaluation and approval of the analyses, the UHPLC-QTOF-MS data were stored in an SQL database (Microsoft SQL server 2019) referred to as ScreenDB.

#### 2.4.2 Retrospective Screening Targets

A list of benzodiazepines was first generated from EMCDDA reports, the database HighResNPS (Mardal et al., 2019), and routine cases. Targets without the measured retention time (RT) were filtered out, as well as common benzodiazepines and metabolites, resulting in a final list of 47 DBZDs. Compound name, molecular formula, RT, molecular mass, fragment ion mass(es), InChIKey, and InChI of the selected DBZDs are presented in Supplementary Table S1.

#### 2.4.3 Retrospective Data Analysis Workflow Training Set and Test Set

A data matrix with m/z-RT hits in the UHPLC-QTOF-MS data file that corresponded to a common benzodiazepine served as the training set. The 13 common benzodiazepines in the training set are presented in Supplementary Table S2. All samples analyzed with UHPLC-QTOF-MS also had paired quantitative results from the UHPLC-MS/MS methods.

The same samples were used for the RDA workflow test set but as a data matrix with m/z-RT hits for DBZDs.

#### 2.4.4 Development of Retrospective Analysis Workflow

The 115,686 extracted features were assigned as true positive or false positive based on the result from the confirmatory LC-MS/MS analysis performed when the cases were open. Precursor, isotopes, and diagnostic fragment ions were extracted from high- and low- energy channel data from ScreenDB with the following limits: ±0.5 min and ±10 mDa from the library value and a count limit of 5. The following variables were tested to set filters for the RDA: count, mass error, retention time, isotope, and diagnostic fragment ions. Isotope ions were calculated as shown in Table 1, and fragment ions were extracted from the in-house screening library. When an m/z-RT hit was detected for a common benzodiazepine in ScreenDB, then fragment and isotope ions were extracted from ScreenDB within an RT window of ±0.05 min and compared with the precursor ion in the low-energy channel (m_0_).

**TABLE 1:** Low- and high-energy channel ions queried in the UHPLC-QTOF-MS data files for each common benzodiazepine [M + H]^+^: protonated molecule.

Low-energy channel ions	
m_0_	[M + H]^+^
m_1_	Isotope with one ^13^C
m_2_	If Br in molecular formula: isotope with one ^81^Br
	If Cl(s) and not Br in molecular formula: isotope with one ^37^Cl
	If halogens not in molecular formula: isotope with two ^13^C
High-energy channel	
f_0_	[M + H]^+^
f_1_	First diagnostic fragment ion
f_2_	Second diagnostic fragment ion
f_3_	Third diagnostic fragment ion
f_4_	Fourth diagnostic fragment ion

#### 2.4.5 Retrospective Screening Workflow

**FIGURE 2:**
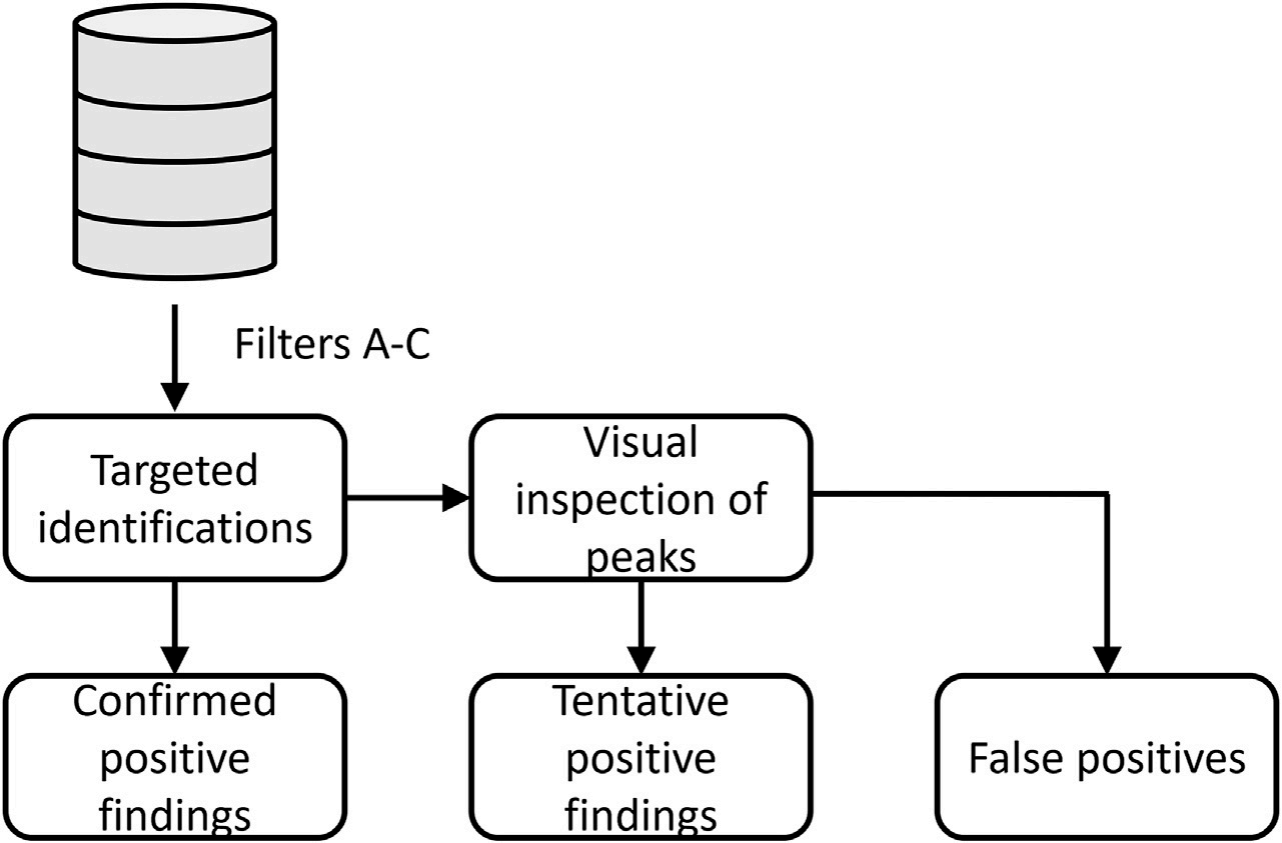
Workflow for classification of designer benzodiazepines identified in driving-under-the-influence of drugs cases.

All data files were processed using UNIFI Scientific Information System (Waters) software and data subsequently parsed to an external SQL database (Microsoft SQL server 2019) with Python (Mollerup et al., 2019). Targets were classified as confirmed positive findings, tentative positive findings, and false-positive (FP) findings as shown in the workflow (Figure 2). First, the targeted identifications were extracted from the SQL database by the following parameters:

A. RT deviation was within 0.25 min between samples
B. Mass difference between measured and theoretical mass was limited to m/z 0.003
C. Intensity threshold of scans was set to 50 counts

If one of the DBZDs was identified in a sample, the case data files were assigned for the retrospective evaluation of DBZDs. Previously targeted identifications, which had been quantified by the UHPLC-MS/MS method, were classified as confirmed positive findings. The remaining data files were reprocessed in UNIFI with an updated library containing all the retrospective screening targets. If the compound was correctly identified by the targeted screening method, then the compound was classified as a tentative positive finding. If targeted identifications were not detected in UNIFI, the extracted ion chromatogram was manually checked for the exact mass. If the target showed a good chromatographic behavior, the compound was also classified as a tentative positive finding. Otherwise, the compound was a false-positive identification. Since the routine screening method in our laboratory is based on a count intensity threshold of 200 in UNIFI, identifications among the tentative findings were analyzed at both 200 and 50 area intensity counts.

## 3 Results

### 3.1 Development of Retrospective Data Analysis Workflow

**FIGURE 3:**
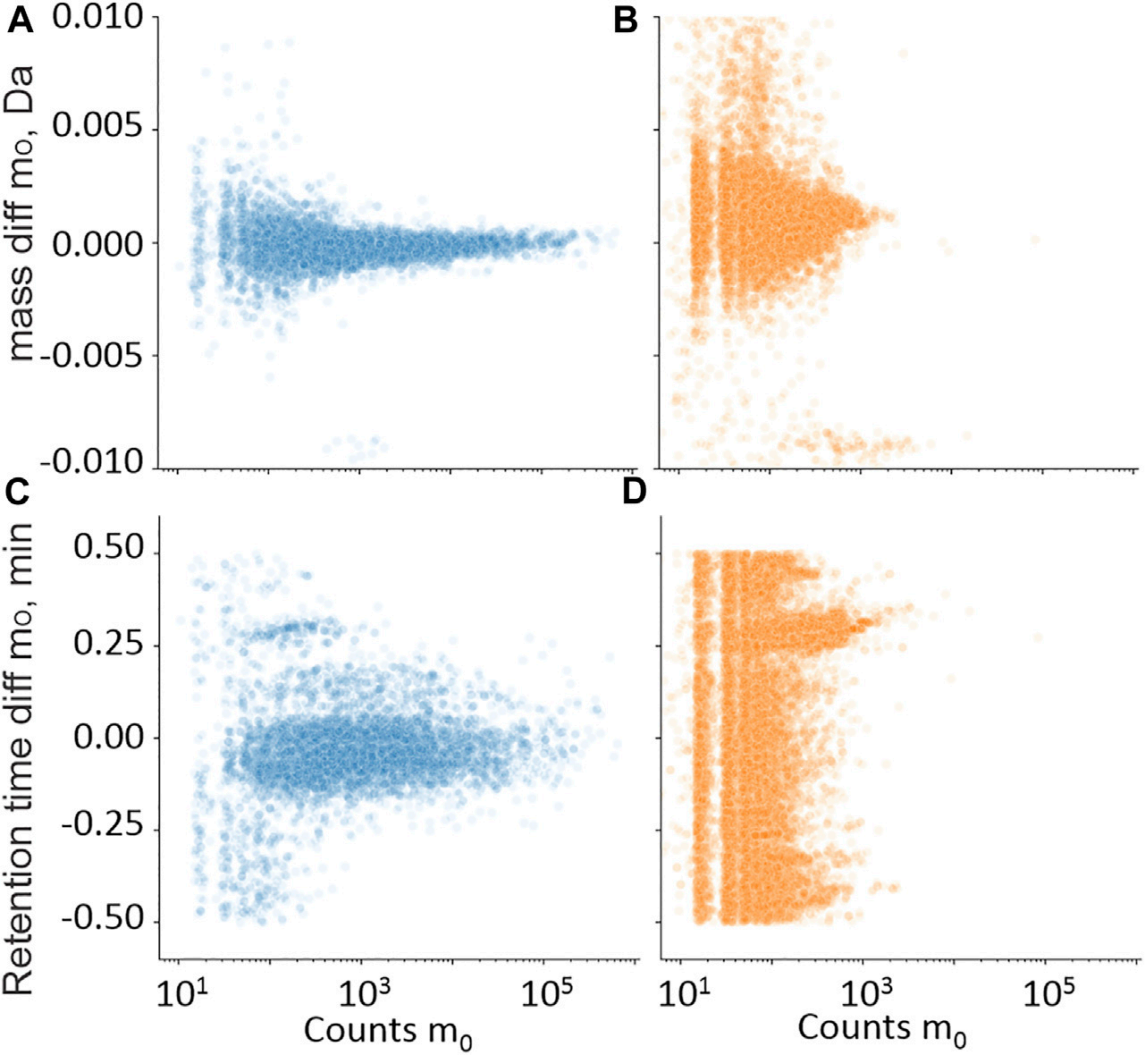
Training set hits for precursor ion m_0_ in the mass **(A,B)** and retention time **(C,D)** domain compared with library values, plotted against m_0_ in the intensity count domain. • Hits from samples with positive quantitative results **(A,C)**; • Hits from samples with negative quantitative results **(B,D)**.

**FIGURE 4:**
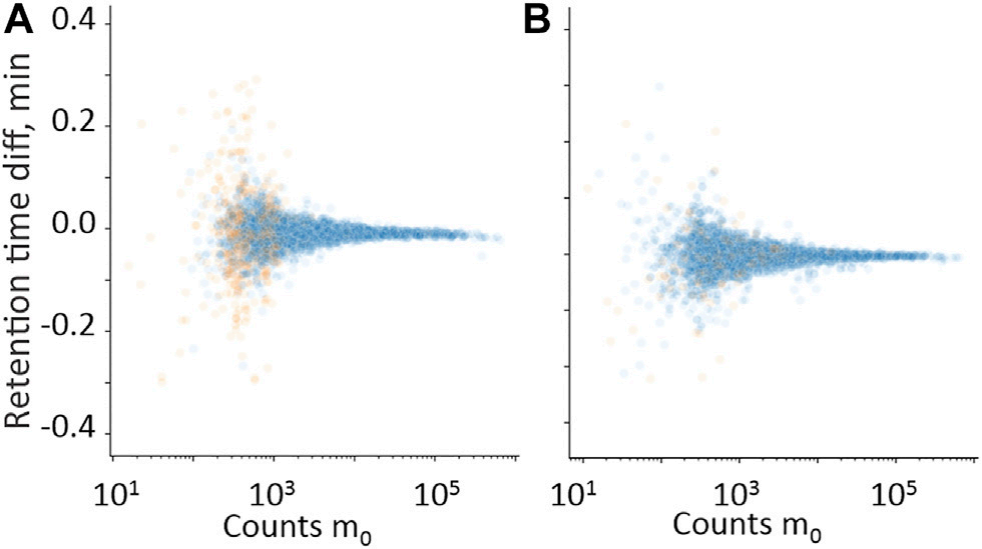
Difference in the retention time domain between measured m_0_ and measured isotopes m_1_ **(A)** and m_2_ **(B)**, plotted against m_0_ in the intensity count domain. • Hits from samples with positive quantitative results; • Hits from samples with negative quantitative results.

**FIGURE 5:**
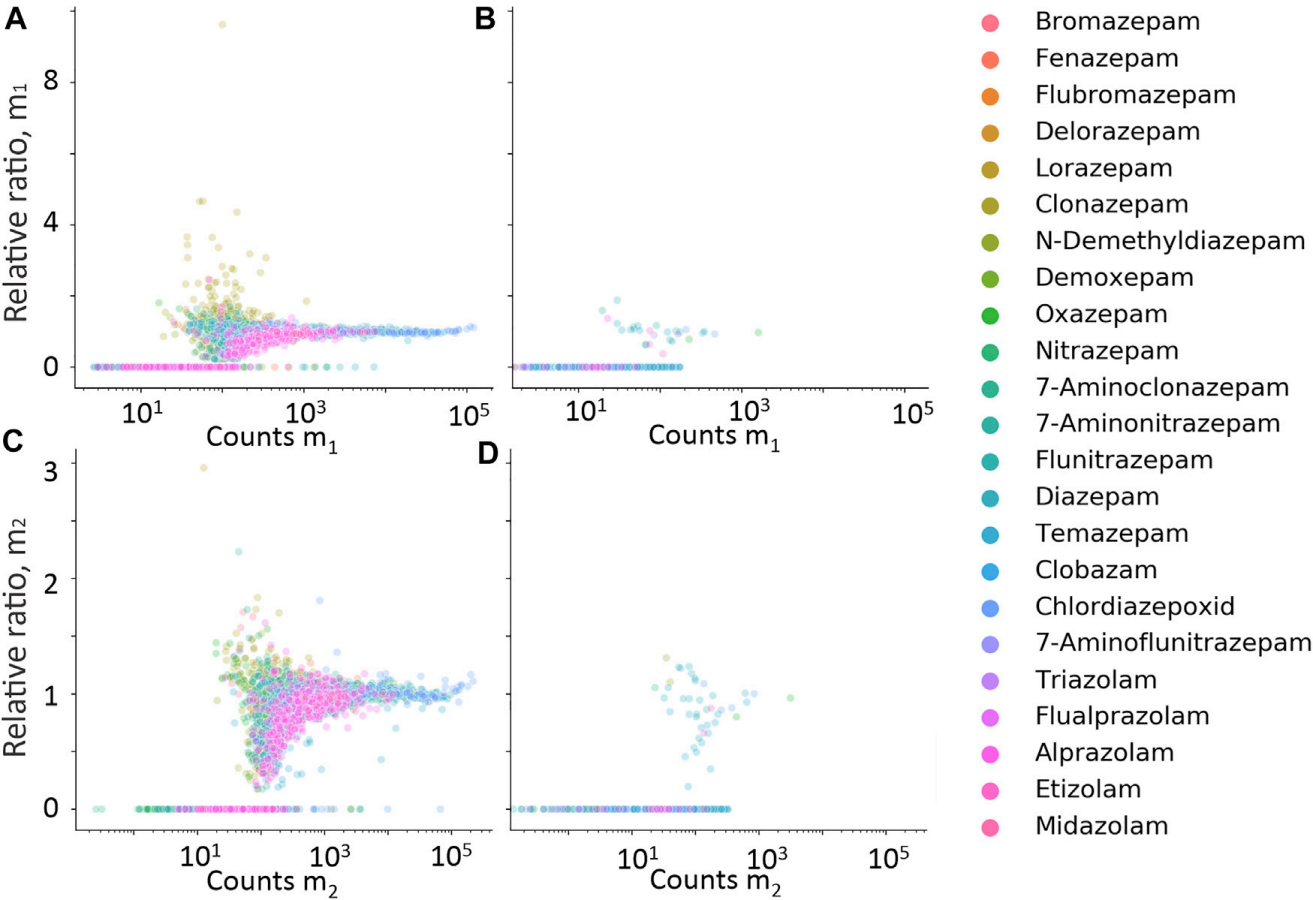
Relative ratio between expected isotope ion counts, and measured isotope ion counts for m_1_ **(A,B)** and m_2_ **(C,D)** plotted against measured counts of m_1_ and m_2_, respectively. Data in **(A,C)** are hits from cases with positive quantitative results, and **(B,D)** from cases with negative quantitative results.

**FIGURE 6:**
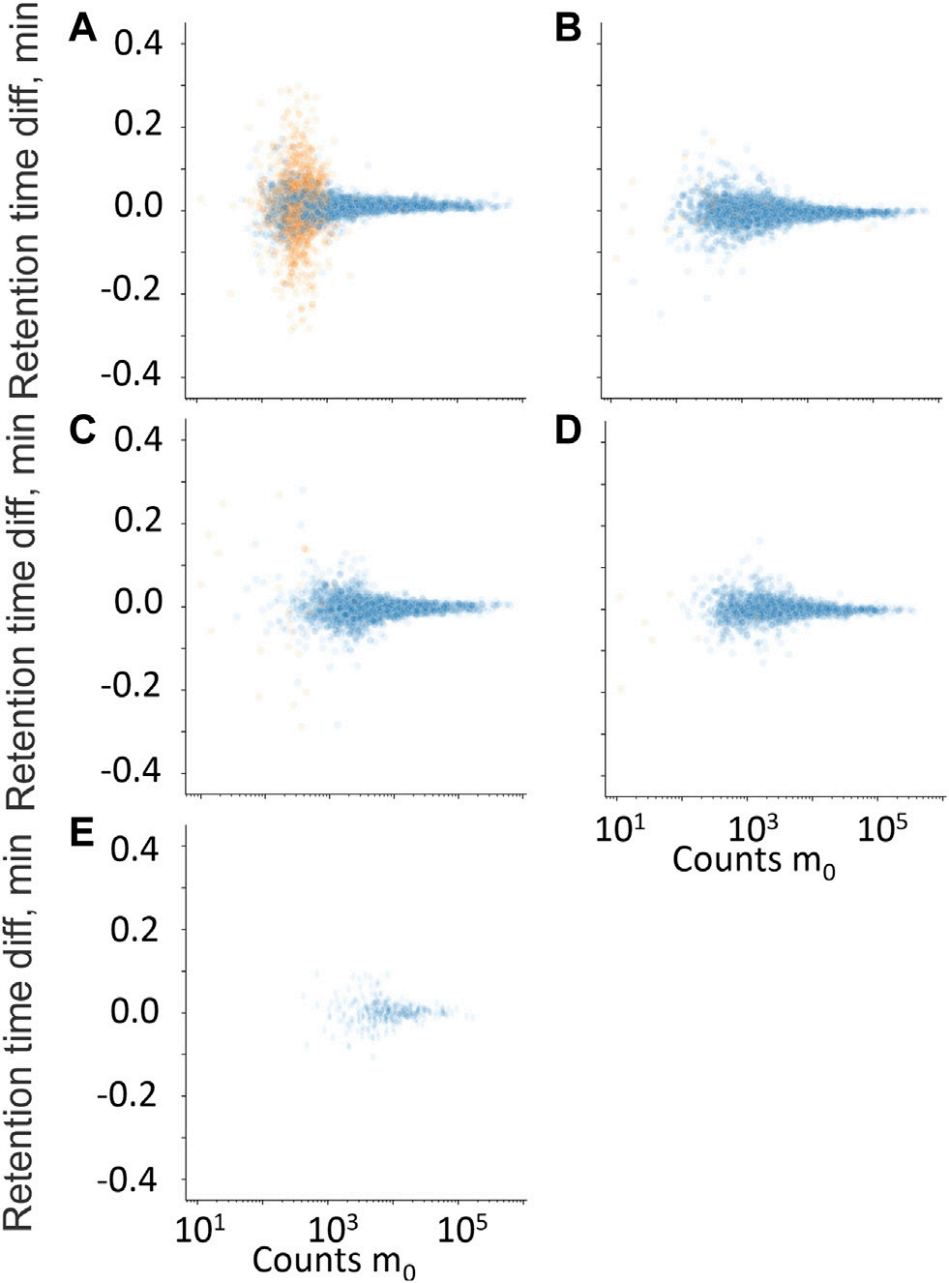
Retention time difference between measured high-energy channel fragment ion and measured m_0_ plotted against m_0_ counts. **(A)** Residual fragment ion, f_0_, **(B)** diagnostic fragment ion f_1_, **(C)** diagnostic fragment ion f_2_, **(D)** diagnostic fragment ion f_3_, and **(E)** diagnostic fragment ion f_4_. • Hits from samples with positive quantitative results; • Hits from samples with negative quantitative results.

**FIGURE 7:**
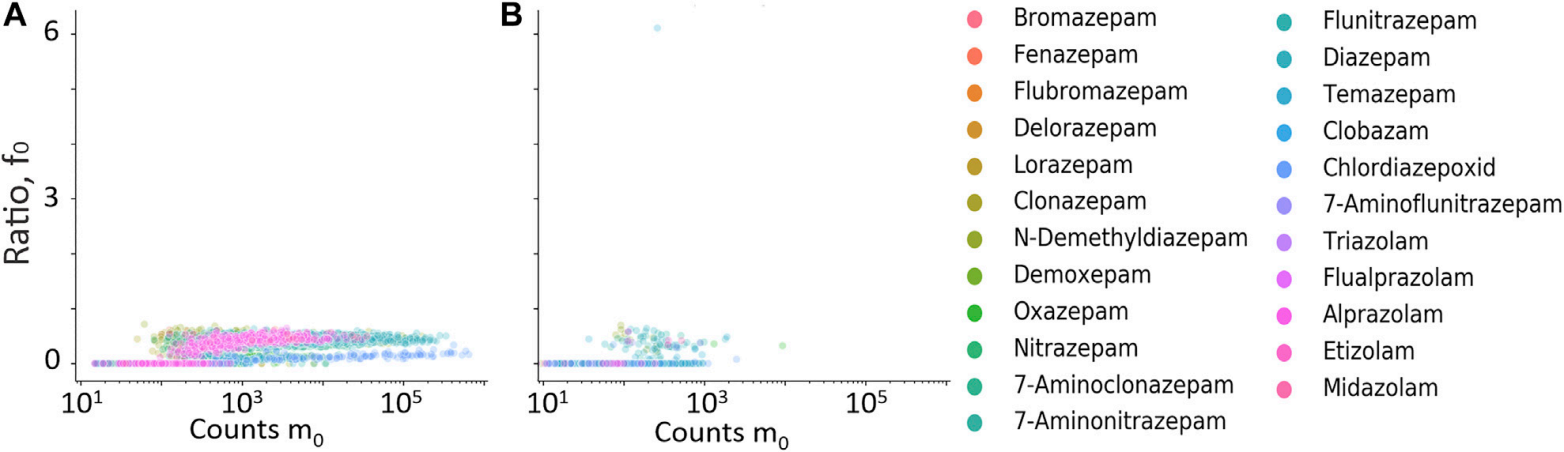
Ratio between residual precursor ion, f_0_, and m_0_ plotted against counts of m_0_. **(A)** Hits from samples with positive quantitative results; **(B)** hits from samples with negative quantitative result.

For the retrospective analysis, historic UHPLC-QTOF-MS screening data from cases also subjected to UHPLC-MS/MS for common benzodiazepines served to evaluate filters for accurate mass, retention time, area count level, fragment ion, and isotope ions. Hits are plotted as m/z-RT pairs, as shown in Figures 3–7, but as analytes may present as multiple hits in a sample, the number of false-positive and true-positive identifications removed by filters in Section 3.1.1 are given in the text.

#### 3.1.1 Mass, Retention Time, and Count Limits

Retention time differences between the measured hits in UHPLC-QTOF-MS were compared with library entries. Figure3A shows the counts of the protonated molecular ion (m_0_) compared with the retention time difference between the measured m_0_ and the library entry for the samples with positive quantitative results of the measured benzodiazepines. Figure3B shows the same plot but where the quantitative result was negative. Reducing the RT limit from 0.5 to 0.25 min removed 1% of the true-positive identifications and 63% of the false-positive identifications.

Data presented in Figures 3A,B were also used to set area count limits. The false positives had generally lower signals than the true positives. Setting the limit to 50 removed 55% of the false positives, but also 2% of the true positive identifications. The same information from the mass domain is presented in Figures 3C, D, with the mass shift in Da on the *y*-axis. Setting the mass limit to 3 mDa filtered out 1% true-positive and 10% false-positive identifications. Combining the mass, retention time, and count limits resulted in the removal of 78% false-positive and 3% true-positive identifications.

#### 3.1.2 Isotope and Fragment Ion Filters

Isotope and fragment ions extracted with a narrower RT window compared with the measured m_0_ were evaluated as additional filters. The retention time difference between m_0_ and isotope ions m_1_ and m_2_ is plotted against counts of m_0_ in Figures 4A, B, respectively. The RT error for isotope ions in true-positive hits is generally less than 0.01 min, which confirms co-elution. The relative ratio of measured counts of m_1_ and m_2_ to the expected counts of m_1_ and m_2,_ plotted against expected counts of the respective isotope is presented in Figures 5A–D for all benzodiazepines detected by UHPLC-QTOF-MS screening and UHPLC-MS/MS. A relative ratio of 1 indicates that the measured isotope count is identical to the count calculated for the isotope based on natural occurrence. At higher expected counts, the isotopes are reliable as filter variables, both in terms of removing false positives and for confirming relative ratios close to 1 for true positives. However, in the lower range of 50–200 counts, the isotope ions are either not detected or the measured ratios deviate more from the calculated ratios. Isotopes are thus not a reliable variable for filters in workflows for lower signals. The count limit was set to 50, so isotopes filters were not used further.

The retention time difference between m_0_ and residual m_0_ in the high-energy spectrum (f_0_), and fragment ions f_1_ through f_4_ is plotted against counts of m_0_ in Figures 6A–E. The fragment ions f_1_ to f_4_ clearly discriminated between true and false positives but mostly at higher counts of m_0_ and were therefore not used further. The residual precursor ion in the high-energy channel (f_0_) was tested as a filter to remove possible false positives that could be in-source fragment ions of structurally similar interferences. Figures 7A, B show the relative ratio between f_0_ and m_0_ plotted against m_0_ counts, for all benzodiazepines detected by UHPLC-QTOF-MS screening and UHPLC-MS/MS. A relative ratio greater than 1 would indicate that the ion in the high-energy channel was higher than that in the low-energy channel. The filter based on the residual precursor ion however only removed one false positive for this group of compounds and will thus not be used in the workflow.

### 3.2 Retrospective Data Analysis for Designer and Uncommon Benzodiazepines

Based on the results from the common benzodiazepines, an RDA workflow was established from filters on the count, retention time, and mass domains.

Of the 47 DBZDs analyzed, the retrospective screening revealed 16 different DBZDs from DUID cases in six years. There were seven confirmed positive compounds reported from the quantified DUID samples, and nine compounds only tentatively identified after the reprocessed data files were checked manually. A large number of hits with low signal intensity were seen in the m/z-RT window corresponding to the DBZD halazepam, which most likely was matrix interferences. A compound-specific count filter was therefore set to count 200 for halazepam. In total, 110 hits were extracted from the ScreenDB with the filters A-C. Metabolites were not considered targets, once their parent drug was detected. Therefore, eight hits of metabolites (delorazepam, *n* = 5; lormetazepam, *n* = 2; and lorazepam, *n* = 1) were excluded as their parent drug (diclazepam) had been confirmed as a positive finding in the same sample. The measured concentrations of metabolites are presented later, even when the parent drug was detected. Chromatographically tailing compounds may present as multiple hits (*n* = 3) but are only counted once. Thus, eleven hits were excluded, and 99 target findings out of 110 hits were further evaluated in our study. Distribution of targets among confirmed positive findings (*n* = 47), tentative positive findings (*n* = 43), and FP findings (*n* = 9) is listed in Table 2.

**TABLE 2:** Designer benzodiazepines (DBZDs) detected by retrospective data analysis in driving-under-the-influence-of-drugs samples from 2014 to 2020 in Eastern Denmark.

Compound	Confirmed positive findings	Tentative positive findings	False positives	Total
Etizolam	18	8		26
Phenazepam	11	2		13
Lorazepam	6	3		9
Flualprazolam	3	5		8
Triazolam	3	4		7
Delorazepam	5	2		7
Tofisopam			6	6
Diclazepam		5		5
Bentazepam			3	3
Flubromazepam	1	1		2
Zolazepam		2		2
Adinazolam		3		3
Flubromazolam		3		3
Clotiazepam		1		1
Quazepam		1		1
Meclonazepam/methylclonazepam		1		1
Estazolam		1		1
Clonazolam		1		1
Total	47	43	9	99

**FIGURE 8:**
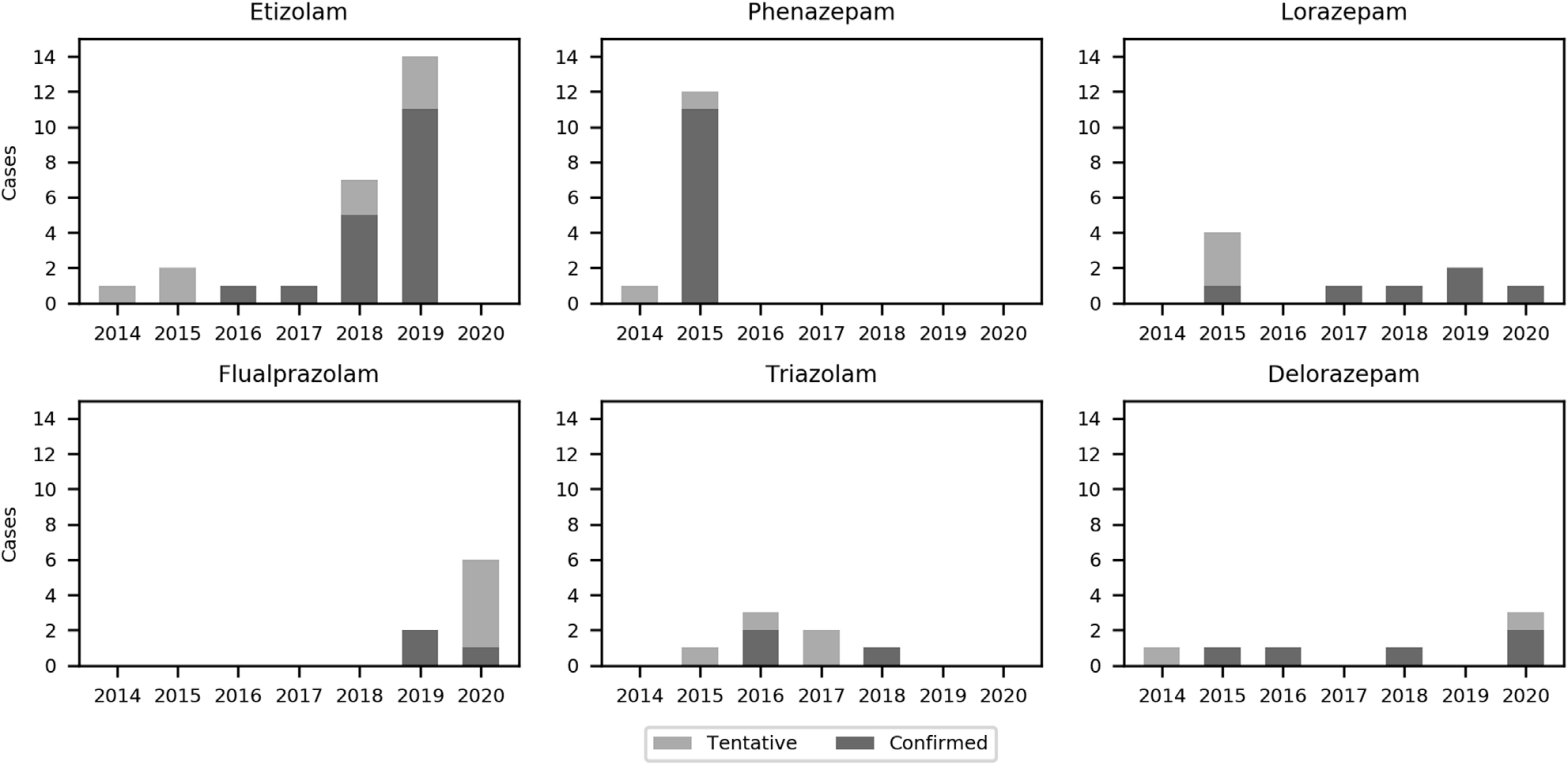
Bar chart of the six most frequently detected designer benzodiazepines in the present study from 2014 to 2020.

The number of confirmed and tentative cases of etizolam, phenazepam, lorazepam, flualprazolam, triazolam, and delorazepam in individual years (2014–2020) is presented in Figure 8. As shown in Supplementary Table S3, seven tentative identifications could only be identified with 50 counts.

#### 3.2.1 Confirmed Positive Findings

Most of the 47 confirmed positive findings were from DBZDs controlled according to the legislation before 2014. Concentrations were given for DBZDs with more than three samples. The median concentrations were 0.013 mg/L for etizolam (*n* = 18), 0.018 mg/L for phenazepam (*n* = 11), 0.046 mg/L for lorazepam (*n* = 6), 0.029 mg/L for delorazepam (*n* = 5), 0.018 mg/L for flualprazolam (*n* = 3), and 0.008 mg/L for triazolam (*n* = 3).

#### 3.2.2 Tentative Positive Findings

Overall, 41 tentative positive findings were identified in UNIFI after updating the library or were manually identified after the visual inspection of peak shapes. There were 23 targets detected in the range 50–200 counts, while 18 targets were detected above the 200 counts. The list of compounds detected at different counts is shown in Supplementary Table S3. The retrospective screening method could not distinguish closely eluting isomers. For example, any hit at *m/z* 330.0640 between 9.15 and 9.59 min could correspond to either meclonazepam or methylclonazepam.

#### 3.2.3 False-Positive Findings

Of the 99 targeted identifications, tofisopam (*n* = 6) and bentazepam (*n* = 3) were categorized as false-positive findings after the visual inspection. For both tofisopam and bentazepam, though, the deviation of RT was within 0.2 min from the expected value and indicated as a positive identification in UNIFI. The hits were categorized as false positives after inspection of the chromatographic peak shape. There were no other DBZDs with identical molecular formula appearing at the RT in the respective sample. The extracted ion chromatogram of these nine targets is included in the supporting information (Supplementary Figure S1).

## 4 Discussion

UHPLC-HRMS-based screening for drugs in biological specimens is usually based on accurate mass, retention time, and fragmentation pattern, possibly with isotopic patterns or adduct filters to rank hits. Accurate mass and fragmentation patterns can be computed or transferred between instruments (Noble et al., 2018; Mardal et al., 2019; Davidsen et al., 2020; Gundersen et al., 2020), but the measured retention time is usually necessary to distinguish among structurally related isomers. In a recent RDA study for NPSs reported by Gundersen et al. (2020), 26 of 43 tentative findings were refuted after the evaluation of the RTs. Therefore, only DBZDs with measured RTs from an analytical standard or pure seizure with a structure confirmed with nuclear magnetic resonance was included in this study. Our laboratory also analyses drug seizures for policy and customs covering the same geographic area, where the DUID cases are collected. It is therefore expected that at least the most relevant DBZD from the collection period is included in the current HR-MS screening library and covered in this study. With the simple filters on count, RT error, and mass tolerances set from the common benzodiazepines, 90 of 99 findings were identified as confirmed or tentative positives. The nine false-positive findings could originate from matrix interference or instrument contamination or noise. For identifying more potential targets, a lower count threshold could be considered. In general screening, increased sensitivity comes with the risk of more false-positive hits. Because of the distinguishable chemical nature of benzodiazepines, decreasing the count threshold to 50 was not a problem for the presented, tailored RDA workflow, and it did allow us for identifying more DBZDs than with a count of 200 (Supplementary Table S3). Halazepam was given a compound-specific count threshold of 200, but since this is a high-dose benzodiazepine that should present with plasma concentrations above 0.01 mg/L 24 h after ingestion (Gupta and Ellinwood, 1990), the RDA workflow should still be able to identify it in DUID cases. At the lower count threshold, however, fragment ions or isotopes could not be used as filters, and using them for ranking of identifications was not necessary. This workflow should not be directly applied to other groups of NPSs as their chemical characteristics are different. Amphetamines or cathinones, which are both higher dosed and have fewer halogens than DBZDs, would require different combinations of the evaluated filters, such as higher count threshold and probably addition of fragment and possibly isotope filters. However, the overall framework presented in this study to tailor RDA workflows to specific groups of compounds can be easily transferred to these other groups of NPSs. It should be noted that of all the groups of NPSs, DBZDs will have the best training set with high chemical homogeneity, and many compounds with several positive identifications.

The three most frequently detected DBZDs in this study, etizolam, flualprazolam, and phenazepam, have all been placed under international control (European Monitoring Centre for Drugs and Drug Addiction (EMCDDA), 2021b). Etizolam and flualprazolam were the two most frequently detected DZBD in numbers and amount seized in the EMCDDA member countries in 2019 and 2020 (European Monitoring Centre for Drugs and Drug Addiction (EMCDDA), 2020). Phenazepam has been detected in a neighboring country to Denmark in 2014 and 2015 (Bäckberg et al., 2019); thus, in the same years, we detected it in our study. Lorazepam is a registered drug in Denmark but was also included in the study, since it is a less frequently prescribed benzodiazepine. Etizolam and phenazepam are not registered drugs in Denmark or EU and are commonly considered as DBZDs by EMCDDA, even though they are legal pharmaceuticals in some other countries (European Monitoring Centre for Drugs and Drug Addiction (EMCDDA), 2021b).

Benzodiazepines have been the most frequently detected class of medicinal drugs in DUID cases, constituting 29–55% of annual cases from 1997 to 2006 in Eastern Denmark (Steentoft et al., 2010), and 12.3% of investigated DUID samples from across Denmark between 2015 and 2019 were positive for a common benzodiazepine (Simonsen et al., 2022). When comparing the 99 DBZDs detected in 13,514 data files in the present study, which is less than 1%, the use of DBZDs compared with conventional benzodiazepines among drivers in the eastern part of Denmark must be considered low. Moreover, of the 99 DBZD identifications, half were already detected and confirmed during the original toxicological evaluation of the DUID case. The results of this study provides epidemiological insights on the extent of DBZD use among drivers in Eastern Denmark and serves as a quality check for the protocols to keep screening methods up-to-date for DBZDs.

Extracting hits from 13,514 LC-HRMS data files with simple count, mass, and retention time filters for 47 compounds was executed in less than 1 min. Prerequisites to apply this workflow in other laboratories are that 1) the main UHPLC-HR-MS analytical method parameters must be kept constant with minor drift in retention time and intensity over time and 2) the raw analytical data are transferred to SQL database, which requires server capacity and some programming experience. A major methodological advancement with the presented framework is thus the speed and efficiency of the data extraction procedure.

## 5 Conclusion

In this study, we presented a framework to develop fast and efficient RDA workflows tailored for groups of NPSs by using historic data for similar compounds. A training set of common benzodiazepines, detected by UHPLC-QTOF-MS and confirmed by LC-MS/MS, formed the basis for setting filters for the subsequent RDA of DBZDs in 13,514 UHPLC-QTOF-MS data files from DUID cases analyzed between 2014 and 2020. Extracting data for 47 DBZDs in 13,514 UHPLC-QTOF-MS data files was executed in less than 1 min, which makes this RDA workflow the most time-efficient of its kind. Etizolam, phenazepam, lorazepam, and flualprazolam were the most frequently detected compounds in the study. We identified 16 DBZDs, corresponding to 90 confirmed or tentative positive findings and nine false positives. The workflow was therefore efficient in extracting DBZD results from many data files. When analytical information on new DBZDs becomes available, the data files can readily be queried again, which makes the strategy useful to detect NPSs in already analyzed forensic samples. The strategy represents an efficient approach to develop tailored workflows also for other groups of NPSs or other exogenous compounds. The study illustrated how scalable data mining studies are possible based on structured, forensic data.

## Data Availability

The data analyzed in this study were subject to the following licenses/restrictions: Data were collected as part of routine forensic investigations. The sensitivity and confidentiality of the data does therefore not allow sharing. Requests to access these datasets should be directed to Marie Mardal, marie.mardal@sund.ku.dk.
