# Supplementary material for: A New Strategy for Efficient Retrospective Data Analyses for Designer Benzodiazepines in Large LC-HRMS Datasets
Source: Front Chem. 2022 May 19;10:868532. doi: 10.3389/fchem.2022.868532 (PMC9175026; doi:10.3389/fchem.2022.868532)
Supplement: Supplementary file 1 [file DataSheet1.docx]

**SI1: Sample preparation:**

General screening by UHPLC-QTOF-MS (Mollerup et al., 2017):

An aliquot of 0.100 g whole blood was treated with 700 μL ACN in a 96‐well plate, shaked and centrifuged at 1000 g for 10 min. Then 50 µL 10% formic acid in ACN was added to an aliquot of 750 µL supernatant before it was evaporated to dryness. The residue was reconstituted in 100 μL of a 25:1:74 mixture of methanol/1% formic acid in water (v/v/v) for the screening method. After another shaking and centrifugation, the extract was transferred to a second 96-well plate and analysed in the UHPLC-QTOF-MS system.

Quantitative analysis for BZD (Hansen et al., 2021):

0.2 g of blank whole blood for calibrators, samples and QCs were weighed individually. All samples were spiked with 20 μL IS solution, and added 800 μL of 1% aqueous formic acid. The deep‐well plate was shaken and centrifuged at 1000*g* for 10 min. 800 μL supernatant from each sample were loaded to pre-conditioned SPE columns (Strata X‐C 30 mg/well, Phenomenex, Torrance, CA, USA). The columns were washed with 900 μL water, then 900 μL 2% formic acid in water containing 5% methanol, and finally with 250 μL methanol. The target analytes were eluted with 2 aliquots of 250 μL of freshly prepared acetonitrile with 8% aqueous ammonia (25%) in water. 100 μL of the eluate was evaporated to dryness under nitrogen at 40 °C and then reconstituted in 200 μL of 50% methanol in water.

Quantitative analysis for DBZD (Mardal et al., 2017):

0.100 g whole blood was mixed with aqueous internal standard and acetonitrile, shaked and centrifuged. The supernatant was added 10% formic acid which was subsequently evaporated to dryness and reconstituted in 100 μL 12.5:12.5:0.05 aqueous methanol:acetonitrile:formic acid (v/v/v).

**SI2: Instrumentation**

UHPLC-QTOF-MS conditions was as previously described(Mollerup et al., 2017), and UHPLC-MS/MS for the quantitative analysis of BZD was as previously described (Hansen et al., 2021).

UHPLC-MS/MS for the quantitative analysis of DBZD was as previously described (Johansen and Jensen, 2005; Mardal et al., 2017) with the following modifications: The analytical column used was an Acquity UPLC® BEH C18 (1.7μm 2.1 x 100 mm). Mobile phase A consisted of 0.05% aqueous formic acid, and Mobile phase B 0.05% formic acid in acetonitrile. The flow rate was 0.6 mL/min, column oven was kept at 50°C, injection volume was 5 μL, and the LC gradient was as follows: Initials conditions with 99.9% A was gradually increased to 95% after 2 minutes, and further to 60% after 6.5 minutes, and 0.1% after 3 minutes where it was kept for 0.5 minutes, and gradually decreased to starting conditions over 0.1 minutes, where it was kept for 1.4 minutes leaving a total run time of 13.5 minutes.

The method had the with the following compound-specific conditions: The MS system was operated with positive electrospray ionization mode (ESI+) with multiple reactions monitoring (MRM) transitions: m/z 305 → 140 for delorazepam and m/z 309 → 140 for delorazepam-d4; m/z 343 → 314 for etizolam and m/z 346 → 317 for etizolam-d3; m/z 327 → 292 for flualprazolam; m/z 373 → 292 for flubromazolam; m/z 314 → 286 for alprazolam-d5 is used for both of the flualprazolam and flubromazolam as IS; m/z 373 → 292 for flubromazolam; m/z 321 → 275 for lorazepam and m/z 327 → 281 for lorazepam-d4; m/z 343 → 239 for triazolam and m/z 347 → 312 for triazolam-d4.

**SI3: Validation parameters for the UHPLC-MS/MS quantitative analysis of DBZD:**

Quality control samples, calibrators, and process efficiency samples for the DBZD for quantitation were run together with the driving-under-the-influence-of-drugs (DUID) blood samples. The calibrators were extracted standards spiked in blank blood, with a minimum of five calibration levels for each quantified analyte, respectively. Lower level of quantitation (LLOQ) ranged from 0.0001 to 0.001 mg/Kg. Spiked blood samples at two concentration levels served as quality control (QC) samples that were run together with the authentic samples, levels are given in Table SI3.1. Standard solutions for QC samples were at prepared from standards from a different vendor or at least diluted from a different stock solution compared with the standard used for calibrators.

Table SI3.1 Validation parameters for the quantitative analysis of DBZD in whole blood samples.

|  | LLOQ [mg/Kg] | ULOQ [mg/Kg] | Calibration levels range [mg/Kg] | QC_low_ [mg/Kg] | QC_high_ [mg/Kg] | QCs analyzed [N] | Average of QC accuracy [%] | Coefficient of vari-ance, QC accuracy |
| --- | --- | --- | --- | --- | --- | --- | --- | --- |
| Delorazepam | 0.001 | 0.25 | 0.001-2 | 0.02 | 0.2/0.25 | 12 | 115.8 | 10.4 |
| Etizolam | 0.0001 | 0.2 | 0.0001-0.2 | 0.0005/  0.01/0.02 | 0.25 | 34 | 100.5 | 4.7 |
| Fenazepam | 0.001 | 0.25 | 0.001-0.25 | 0.02/  0.095 | 0.3 | 18 | 103.9 | 11.3 |
| Flualprazolam | 0.0005 | 0.2 | 0.0005-0.2 | 0.002 | 0.025 | 4 | 97.1 | 1.2 |
| Lorazepam | 0.001 | 2 | 0.001-2 | 0.095 | 0.2/0.3 | 21 | 110.3 | 12.0 |
| Triazolam | 0.001 | 0.25 | 0.001-0.25 | 0.02/  0.095 | 0.2/0.3 | 8 | 115.6 | 10.6 |

LLOQ: lower limit of quantitation, QC: quality control, ULOQ: upper limit of quantitation

Matrix effect (ME), extraction recovery (ER), and process efficiency (PE) were evaluated by the three-set approach described by Matuszewski (Matuszewski et al., 2003) when a DBZD was included in an analytical run. In the first set, samples were spiked with all analytes before extraction, and in the second set, the samples were spiked after extraction. The third set consisted of a pure standard solution. The ME, expressed as a percentage, was calculated by dividing the IS-corrected peak area in samples spiked after extraction (second set) by the IS-corrected peak area in pure standard solution (third set). The ER was calculated by dividing the IS-corrected peak areas in samples spiked before extraction (first set) by the IS-corrected peak area in samples spiked after extraction (second set). The PE (%) was calculated by dividing the IS-corrected peak area in samples spiked before extraction (first set) by the IS-corrected peak area in pure standard solution (third set). ME, ER, and PE were tested at two concentration levels for all analytes in blank blood samples. All samples were spiked with ISs before extraction. Results for ER, ME, and PE are given in Table SI3.2.

Table SI3.2

|  |  | Extraction recovery | | | Matrix effect | | | Process eficiency | | |
| --- | --- | --- | --- | --- | --- | --- | --- | --- | --- | --- |
|  | Tests (N) | min | average | max | min | average | max | min | average | max |
| Delorazepam | 10 | 65 | 74 | 79 | 87 | 103 | 117 | 56 | 76 | 84 |
| Etizolam | 32 | 56 | 74 | 89 | 102 | 116 | 152 | 69 | 85 | 113 |
| Fenazepam | 11 | 56 | 72 | 86 | 88 | 105 | 135 | 62 | 76 | 92 |
| Flualprazolam | 4 | 63 | 71 | 79 | 98 | 104 | 114 | 64 | 74 | 78 |
| Lorazepam | 12 | 59 | 70 | 75 | 96 | 109 | 119 | 65 | 76 | 87 |
| Triazolam | 6 | 69 | 72 | 74 | 97 | 104 | 110 | 72 | 75 | 78 |

Table S1. Compounds name, molecular formula, retention time (RT), molecular mass, fragments mass, InChIKey of designer benzodiazepine targets used for retrospective data analysis.

| **name** | **formula** | **Rt [min]** | **m0** | **m1** | **m2** | **f1** | **f2** | **f3** | **f4** | InChIkey |
| --- | --- | --- | --- | --- | --- | --- | --- | --- | --- | --- |
| 2-Hydroxyethylflurazepam | C17H14ClFN2O2 | 8.6 | 333.0801 | 334.0832 | 335.0777 |  |  |  |  | FOCBRQQHNOKOJQ-UHFFFAOYSA-N |
| 3-Hydroxybromazepam | C14H10BrN3O2 | 5.3 | 332.0029 | 333.0059 | 334.0010 | 286.9815 |  |  |  | URRUSNGCYBXNLO-UHFFFAOYSA-N |
| 3-Hydroxyphenazepam | C15H10BrClN2O2 | 8.4 | 364.9687 | 365.9718 | 366.9665 | 318.9632 | 346.9581 | 273.0022 |  | KRJKJUWAZOWXNV-UHFFFAOYSA-N |
| 4-Chlorodiazepam | C16H12Cl2N2O | 11.5 | 319.0399 | 320.0431 | 321.0372 | 154.0418 | 227.0496 | 256.0762 |  | PUMYFTJOWAJIKF-UHFFFAOYSA-N |
| 4-Hydroxyclobazam | C16H13ClN2O3 | 6.7 | 317.0687 | 318.0719 | 319.0664 | 275.0583 |  |  |  | NKSXJAYACJZMBM-UHFFFAOYSA-N |
| 4-Hydroxytriazolam | C17H12Cl2N4O | 7.7 | 359.0461 | 360.049 | 361.0434 | 341.0355 | 314.0246 | 272.9981 |  | BHUYWUDMVCLHND-UHFFFAOYSA-N |
| Adinazolam | C19H18ClN5 | 5.6 | 352.1323 | 353.1352 | 354.1300 | 58.0651 | 295.0745 | 267.0558 |  | GJSLOMWRLALDCT-UHFFFAOYSA-N |
| alpha-Hydroxyalprazolam | C17H13ClN4O | 7.8 | 325.0851 | 326.0880 | 327.0827 | 307.0723 |  |  |  | ZURUZYHEEMDQBU-UHFFFAOYSA-N |
| alpha-Hydroxytriazolam | C17H12Cl2N4O | 7.6 | 359.0461 | 360.0490 | 361.0434 | 331.0274 | 341.0355 | 313.0294 | 176.0267 | BHUYWUDMVCLHND-UHFFFAOYSA-N |
| Bentazepam | C17H16N2OS | 6.5 | 297.1056 | 298.1086 | 299.1047 | 166.0685 | 269.1107 | 241.0794 | 210.1277 | AIZFEOPQVZBNGH-UHFFFAOYSA-N |
| Bromazolam | C17H13BrN4 | 8.7 | 353.0396 | 354.0426 | 355.0377 | 325.0209 | 274.1213 | 205.0760 |  | KCEIOBKDDQAYCM-UHFFFAOYSA-N |
| Brotizolam | C15H10BrClN4S | 9.2 | 392.9571 | 393.9598 | 394.9548 | 314.0388 |  |  |  | UMSGKTJDUHERQW-UHFFFAOYSA-N |
| Clotiazepam | C16H15ClN2OS | 11.1 | 319.0666 | 320.0696 | 321.0640 | 291.0710 | 154.0682 | 218.0727 | 74.0064 | CHBRHODLKOZEPZ-UHFFFAOYSA-N |
| Cloxazolam | C17H14Cl2N2O2 | 4.1 | 349.0505 | 350.0537 | 351.0479 |  |  |  |  | ZIXNZOBDFKSQTC-UHFFFAOYSA-N |
| Delorazepam | C15H10Cl2N2O | 9.4 | 305.0243 | 306.0274 | 307.0216 | 140.0262 | 165.0214 |  |  | CHIFCDOIPRCHCF-UHFFFAOYSA-N |
| Desalkylflurazepam | C15H10ClFN2O | 8.9 | 289.0538 | 290.0570 | 291.0513 | 140.0262 | 226.0901 |  |  | UVCOILFBWYKHHB-UHFFFAOYSA-N |
| Deschloroetizolam | C17H16N4S | 8.4 | 309.1168 | 310.1196 | 311.1158 | 255.0950 | 280.0777 | 225.0607 | 239.0637 | JIOBORXCOGMHSV-UHFFFAOYSA-N |
| Diclazepam | C16H12Cl2N2O | 10.8 | 319.0399 | 320.0431 | 321.0372 | 227.0496 | 154.0418 | 256.0762 | 291.0450 | VPAYQWRBBOGGPY-UHFFFAOYSA-N |
| Estazolam | C16H11ClN4 | 8.1 | 295.0745 | 296.0774 | 297.0720 | 267.0558 | 205.0761 |  |  | CDCHDCWJMGXXRH-UHFFFAOYSA-N |
| Ethyl loflazepate | C18H14ClFN2O3 | 11.0 | 361.0750 | 362.0781 | 363.0727 | 259.0428 | 287.0376 | 289.0532 | 166.0052 | CUCHJCMWNFEYOM-UHFFFAOYSA-N |
| Etizolam | C17H15ClN4S | 9.2 | 343.0779 | 344.0807 | 345.0752 | 314.0387 | 259.0217 | 206.0746 | 293.0855 | VMZUTJCNQWMAGF-UHFFFAOYSA-N |
| Phenazepam | C15H10BrClN2O | 9.7 | 348.9738 | 349.9769 | 350.9716 | 206.0830 | 183.975 | 242.0598 |  | MPZVLJCMGPYWQQ-UHFFFAOYSA-N |
| Flualprazolam | C17H12ClFN4 | 8.2 | 327.0807 | 328.0836 | 329.0783 | 292.1119 | 299.0620 |  |  | ZRKDDZBVSZLOFS-UHFFFAOYSA-N |
| Flubromazepam | C15H10BrFN2O | 9.2 | 333.0033 | 334.0064 | 335.0014 | 226.0901 | 183.9756 | 179.0730 | 208.9709 | APTIFBSTEIXFOB-UHFFFAOYSA-N |
| Flubromazepam isomer | C15H10BrFN2O | 8.4 | 333.0033 | 334.0064 | 335.0014 | 225.0823 | 198.0714 | 253.0772 | 124.0557 | VXGSZBZQCBNUIP-UHFFFAOYSA-N |
| Flubromazolam | C17H12BrFN4 | 8.5 | 371.0302 | 372.0331 | 373.0283 | 292.1119 | 343.0115 | 263.0979 | 171.0791 | PPTYJKAXVCCBDU-UHFFFAOYSA-N |
| Flunitrazolam | C17H12FN5O2 | 6.9 | 338.1048 | 339.1076 | 340.1102 | 292.1119 | 264.0932 | 310.0861 |  | SAADBVWGJQAEFS-UHFFFAOYSA-N |
| Flurazepam | C21H23ClFN3O | 6.7 | 388.1586 | 389.1617 | 390.1565 | 315.0695 | 288.0586 | 225.0949 |  | WYCLKVQLVUQKNZ-UHFFFAOYSA-N |
| Halazepam | C17H12ClF3N2O | 11.4 | 353.0663 | 354.0694 | 355.0639 | 241.0528 |  |  |  | UTEFBSAVJNEPTR-RGEXLXHISA-N |
| Loprazolam | C23H21ClN6O3 | 6.3 | 465.1436 | 466.1465 | 467.1417 | 85.0764 | 111.0918 | 408.0849 | 70.0656 | DIWRORZWFLOCLC-UHFFFAOYSA-N |
| Lorazepam | C15H10Cl2N2O2 | 8.3 | 321.0192 | 322.0223 | 323.0165 | 275.0138 | 229.0528 | 138.0106 |  | FJIKWRGCXUCUIG-UHFFFAOYSA-N |
| Lormetazepam | C16H12Cl2N2O2 | 9.6 | 335.0349 | 336.0380 | 337.0322 | 289.0294 | 243.0684 | 177.0215 | 317.0243 | LMUVYJCAFWGNSY-UHFFFAOYSA-N |
| Meclonazepam | C16H12ClN3O3 | 9.3 | 330.0640 | 331.0670 | 332.0616 | 284.0711 | 239.0496 | 214.0418 | 204.0808 | YLCXGBZIZBEVPZ-UHFFFAOYSA-N |
| Medazepam | C16H15ClN2 | 6.7 | 271.0997 | 272.1028 | 273.0971 | 242.0732 | 207.1043 | 180.0449 |  | AZVBJJDUDXZLTM-UHFFFAOYSA-N |
| Methylclonazepam | C16H12ClN3O3 | 9.4 | 330.0640 | 331.0670 | 332.0616 | 284.0711 |  |  |  | NQSSWDKQLVBUQN-UHFFFAOYSA-N |
| Midazolam | C18H13ClFN3 | 6.6 | 326.0855 | 327.0885 | 328.0831 | 291.1167 | 244.0324 | 209.0636 |  | AKPLHCDWDRPJGD-UHFFFAOYSA-N |
| Nifoxipam | C15H10FN3O4 | 6.3 | 316.0728 | 317.0758 | 318.0782 | 270.0673 | 298.0622 | 194.0838 | 260.0830 | GWUSZQUVEVMBPI-UHFFFAOYSA-N |
| Nimetazepam | C16H13N3O3 | 8.9 | 296.1030 | 297.106 | 298.1085 | 250.1101 | 221.1073 | 268.1081 | 193.0886 | KJONHKAYOJNZEC-UHFFFAOYSA-N |
| Norchlordiazepoxide | C15H12ClN3O | 3.5 | 286.0742 | 287.0772 | 288.0716 |  |  |  |  | ADIMAYPTOBDMTL-UHFFFAOYSA-N |
| Prazepam | C19H17ClN2O | 11.6 | 325.1102 | 326.1134 | 327.1079 | 271.0633 | 140.0262 |  |  | MWQCHHACWWAQLJ-UHFFFAOYSA-N |
| Pyrazolam | C16H12BrN5 | 5.9 | 354.0349 | 355.0377 | 356.0330 | 206.0838 | 167.073 | 205.0760 | 275.1165 | BGRWSFIQQPVEML-UHFFFAOYSA-N |
| Quazepam | C17H11ClF4N2S | 11.6 | 387.0340 | 388.0370 | 389.0314 | 313.0276 | 354.0542 |  |  | IKMPWMZBZSAONZ-UHFFFAOYSA-N |
| Tetrazepam | C16H17ClN2O | 11.0 | 289.1102 | 290.1134 | 291.1077 | 253.1336 |  |  |  | IQWYAQCHYZHJOS-UHFFFAOYSA-N |
| Tofisopam | C22H26N2O4 | 9.3 | 383.1965 | 384.1997 | 385.2025 | 298.1074 | 313.1309 | 342.1700 |  | RUJBDQSFYCKFAA-UHFFFAOYSA-N |
| Triazolam | C17H12Cl2N4 | 8.7 | 343.0512 | 344.0541 | 345.0485 | 308.0824 | 315.0325 |  |  | JOFWLTCLBGQGBO-UHFFFAOYSA-N |
| Zolazepam | C15H15FN4O | 3.1 | 287.1303 | 288.1331 | 289.1359 | 271.0998 | 243.1046 | 230.1089 | 162.0356 | GDSCFOSHSOWNDL-UHFFFAOYSA-N |

Tabel S2

| List of common benzodiazepine targets used in training set: |
| --- |
| Chlordiazepoxid |
| Diazepam |
| Nordazepam/N-demethyldiazepam |
| Temazepam |
| Oxazepam |
| Alprazolam |
| Bromazepam |
| Nitrazepam |
| 7-Aminonitrazepam |
| Clonazepam |
| 7-Aminoclonazepam |
| Flunitrazepam |
| 7-Aminoflunitrazepam |

| Table S3. Tentative positive findings with 50 counts and 200 counts. | | | |
| --- | --- | --- | --- |
|  | Intensity threshold | |  |
| Compound | 50 Counts | 200 Counts | Total |
| Etizolam | 4 | 4 | 8 |
| Diclazepam | 2 | 3 | 5 |
| Flualprazolam |  | 5 | 5 |
| Triazolam | 3 | 1 | 4 |
| Lorazepam | 3 |  | 3 |
| Adinazolam | 3 |  | 3 |
| Delorazepam | 1 | 1 | 2 |
| Flubromazolam | 2 |  | 2 |
| Phenazepam |  | 2 | 2 |
| Zolazepam | 2 |  | 2 |
| Clonazolam |  | 1 | 1 |
| Estazolam |  | 1 | 1 |
| Clotiazepam | 2 |  | 2 |
| Flubromazepam | 1 |  | 1 |
| Meclonazepam/Methylclonazepam | 1 |  | 1 |
| Quazepam | 1 |  | 1 |
| Total | 25 | 18 | 43 |

Fig. S1: Extracted ion chromatograms of hits categorized as false negative identifications for Tofisopam (A) and Bentazepam (B).


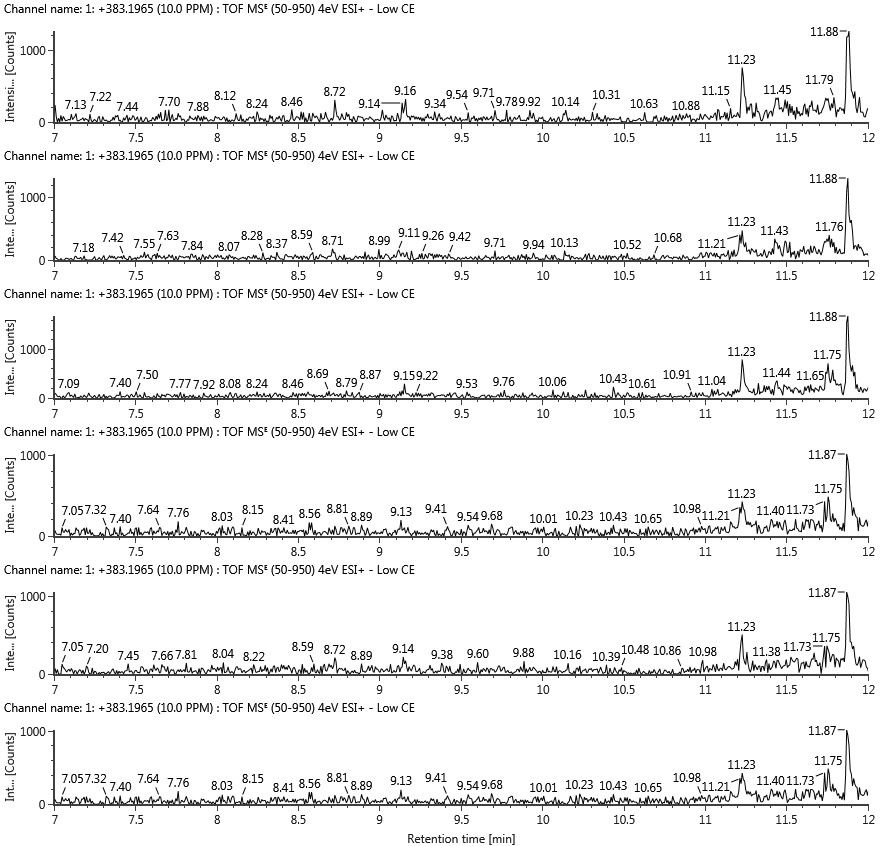


A. Tofisopam


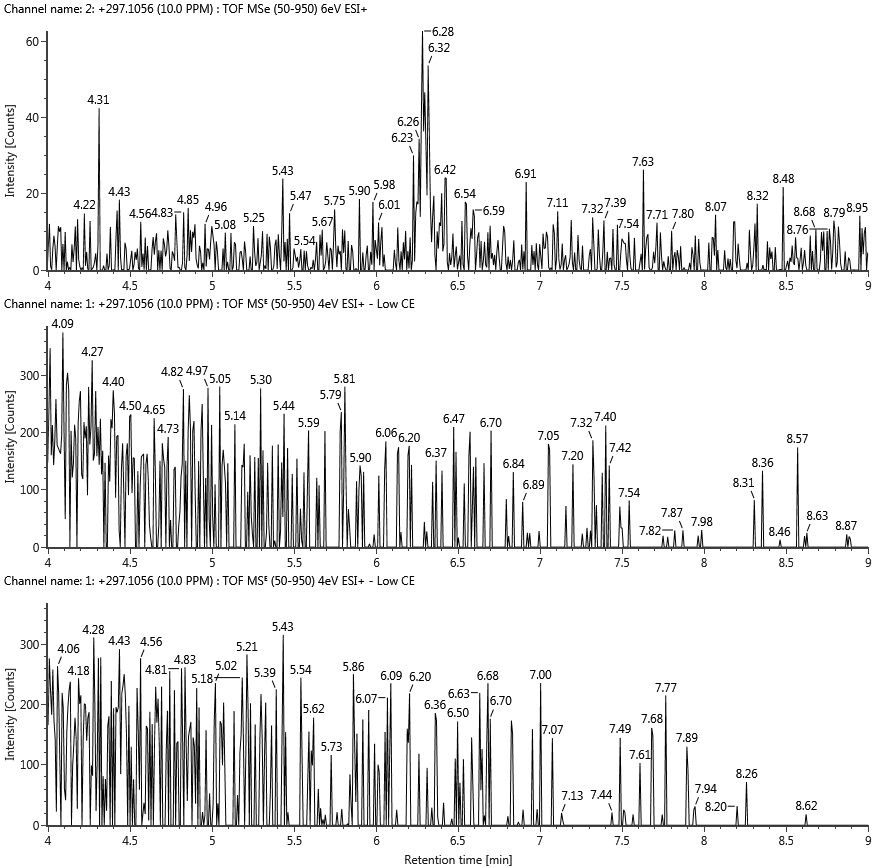


B. Bentazepam

Hansen, S. L., Nielsen, M. K. K., Linnet, K., and Rasmussen, B. S. (2021). Simple implementation of muscle tissue into routine workflow of blood analysis in forensic cases – A validated method for quantification of 29 drugs in postmortem blood and muscle samples by UHPLC–MS/MS. *Forensic Sci. Int.* 325. doi:10.1016/J.FORSCIINT.2021.110901.

Johansen, S. S., and Jensen, J. L. (2005). Liquid chromatography-tandem mass spectrometry determination of LSD, ISO-LSD, and the main metabolite 2-oxo-3-hydroxy-LSD in forensic samples and application in a forensic case. *J. Chromatogr. B Anal. Technol. Biomed. Life Sci.* 825, 21–28. doi:10.1016/j.jchromb.2004.12.040.

Mardal, M., Johansen, S. S., Thomsen, R., and Linnet, K. (2017). Advantages of analyzing postmortem brain samples in routine forensic drug screening—Case series of three non-natural deaths tested positive for lysergic acid diethylamide (LSD). *Forensic Sci. Int.* 278, e14–e18. doi:10.1016/j.forsciint.2017.07.025.

Matuszewski, B. K., Constanzer, M. L., and Chavez-Eng, C. M. (2003). Strategies for the assessment of matrix effect in quantitative bioanalytical methods based on HPLC-MS/MS. *Anal. Chem.* 75, 3019–3030. doi:10.1021/ac020361s.

Mollerup, C. B., Dalsgaard, P. W., Mardal, M., and Linnet, K. (2017). Targeted and non-targeted drug screening in whole blood by UHPLC-TOF-MS with data-independent acquisition. *Drug Test. Anal.* 9, 1052–1061. doi:10.1002/dta.2120.
